# HIV prevalence, related risk behaviors, and correlates of HIV infection among people who use drugs in Cambodia

**DOI:** 10.1186/s12879-018-3472-3

**Published:** 2018-11-13

**Authors:** Heng Sopheab, Chhorvann Chhea, Sovannary Tuot, Jonathan A. Muir

**Affiliations:** 1 School of Public Health at the National Institute of Public Health, Lot #80, Samdech Penn Nouth Blvd. Tuol Kork District, Phnom Penh, Cambodia; 2 Center for Population and Health Research, KHANA, Phnom Penh, Cambodia; 3 Department of Epidemiology, University of Washington, Seattle, USA

**Keywords:** HIV, People who use drugs, People who inject drugs, PWID, Non-PWID, ATS users, Cambodia

## Abstract

**Background:**

Although HIV prevalence in Cambodia has declined to 0.6% among the general population, the prevalence remains high among female sex workers (14.0%) and men who have sex with men (2.3%). Over the past 10 years, the number of people who use drugs (PWUDs) has increased considerably. PWUDs, especially people who inject drugs (PWIDs), who have multiple sex partners or unprotected sex contribute to a higher HIV prevalence. This paper aims to estimate the prevalence of HIV across PWUD groups and to identify factors associated with HIV infection.

**Methods:**

Respondent-driven sampling (RDS) was used to recruit 1626 consenting PWUDs in 9 provinces in 2012. Questionnaires and blood specimens were collected. HIV prevalence estimates were calculated using RDSAT 7.1. Individual weightings for HIV were generated with RDSAT and used for a weighted analysis in STATA 13. Multivariate logistic regression was used to identify the independent factors associated with HIV prevalence.

**Results:**

Most of the PWUDs were men (82.0%), and 7.3% were PWIDs. Non-PWIDs, especially users of amphetamine-type stimulants (ATS), represented the larger proportion of the participants (81.5%). The median age for of the PWUDs was 24.0 years (IQR: 20–29). The HIV prevalence among the PWUDs was 5.1% (95% CI: 4.1–6.2), 24.8%, among PWIDs and 4.0% among non-PWIDs. The HIV prevalence among female PWIDs was 37.5, and 22.5% among male PWIDs. Four factors were independently associated with HIV infection: female sex, with AOR = 7.8 (95% CI: 3.00–20.35); age groups 21–29 and older (AOR = 10.3, 95% CI: 1.2–20.4); and using drugs for ≥12 months (AOR = 4.0, 95% CI: 1.38–11.35). Finally, injecting drugs remained a strong predictor of HIV infection, with an AOR = 4.1 (95% CI: 1.53–10.96).

**Conclusion:**

HIV prevalence remains high among PWIDs. Harm reduction efforts, such as needle and syringe provision programs, must improve their coverage. Innovative strategies are needed to reach sub-groups of PWUDs, especially women who inject drugs. Furthermore, the large proportion of non-PWIDs, especially ATS users, should not be ignored. Therefore, combined HIV prevention and harm reduction programs should integrate ATS users.

## Background

Over the past 25 years, remarkable progress has been made in the fight against HIV in Cambodia. The HIV prevalence has fallen from 2.0% (1999) to 0.6% (2015) among the general population aged 15–49 years [1]. However, a high prevalence is still observed among key populations, such as female sex workers (14.0 to 15.0%) [2, 3] and men who have sex with men (2.3%) [4].

Since 2004, evidence has indicated an increase in the number of people who use drugs (PWUD) and the availability of illicit drugs in Cambodia. The country has changed from a drug trafficking transit location to a site of drug production and use [5, 6]. The country has been affected by illicit drug abuse problems, mainly amphetamine-type stimulant (ATS) use. Notable increases have been observed among youth and sex workers. A study among youth out of school in 2010 showed that approximately 4% of young women and 15% of young men aged 10 to 24 years reported ever having used drugs [7].

PWUDs, especially people who inject drugs (PWIDs) who share syringes and needles with multiple partners or have unprotected sex contribute considerably to a higher HIV prevalence. Non-injecting drug users are at a higher risk of experiencing physical and mental health problems [8] and poly-substance abuse disorder. They also may become injecting drug users with an increased risk of HIV infection [9–11]. The 2012 estimate of the size of this key population, conducted by the National Center for HIV/AIDS, Dermatology and STIs (NCHADS), suggested that there were approximately 28,000 PWUDs in Cambodia; half of them were ATS users, and close to 7% reported injecting heroin [12].

In Asia, the HIV prevalence among PWIDs varies from country to country and within countries. For example, the HIV prevalence among PWIDs was 36.4% in Indonesia (2011), 25.2% in Thailand, 16.6% in Malaysia, 10.5% in Vietnam, and 6.4% in China [13, 14]. Factors that may account for this variability between countries include the intensity of harm reduction programs, overlapping risk behaviors (e.g. interaction with paid sex, and having multiple sex partners) and drug injection and social and sexual networking [15, 16].

In the past, studies have indicated that factors associated with the HIV prevalence among PWIDs include socio-demographic characteristics (e.g., sex, older age, marital status, less education), risky sexual behaviors (e.g., paid sex, sex exchanged for drugs) and risky behaviors during drug use (e.g., needle and syringe sharing, using injected drugs for more than one year) [14, 17, 18].

In Cambodia, there have been a few studies on drug use policy and harm reduction intervention programs, such as needle and syringe distribution programs [19, 20]. For example, Chheng et al indicated that in 2003, the Government of Cambodia acknowledged the importance and necessity of harm reduction approaches to prevent HIV transmission among PWUDs and their sexual partners. However, the harm reduction intervention was never fully implemented due to limited awareness and support from law enforcement at the local level as well as budgeting commitment [19]. The failure of this policy indeed had a negative impact on HIV prevention and harm reduction for the highest-risk groups, such as PWUDs.

Little is known about the characteristics and patterns of drug use in Cambodia. Moreover, the HIV prevalence among this key population has never been estimated nationwide. Therefore, this paper, which used data from a study conducted in late 2012–2013 [12], sought to estimate the prevalence of HIV infection among people who inject drugs (PWIDs) and drug users who do not inject drugs (non-PWIDs), and examine factors associated with HIV infection in these populations.

## Methods

### Study sites and population

Nine provinces were purposively selected for the study: Phnom Penh, Sihanoukville, Kampong Speu, Battambang, Banteay Meanchey, Siem Reap, Kampong Cham, Prey Veng, and Svay Rieng. These provinces were selected based on a program report indicating that they accounted for 85% of all PWUDs in Cambodia and that drug abuse activity in these provinces was significantly high *(Consultative Technical Working Group on Drug and HIVAIDS, 2012)*. Study participants were individuals at least 15 years old who reported using any illicit drug, including heroin, cocaine, opiates, amphetamines, methamphetamines, yama, ice, crystal and ketamine, in the past 12 months.

In the analysis, the 9 provinces were separated into two groups based on the HIV prevalence among PWUDs: those with an HIV prevalence ≥4% were assigned to the high-risk province group (Sihanoukville, Phnom Penh, Battambang, and Banteay Meanchey), and those with a lower prevalence were assigned to the low-risk province group (Kampong Speu, Kampong Cham, Prey Veng, Svay Rieng and Siem Reap).

### Sampling and sample size

We used respondent-driven sampling (RDS), a network referral method, to recruit this hard-to-reach population (PWUDs) to ensure a representative sample [21]. At the beginning, 36 diverse seeds (4 seeds per province) were recruited through local NGOs working with drug users. They were selected based on sex (male/female) and type of drug use (injecting/non-injecting). Each seed was asked to recruit other 2 eligible PWUDs from their personal network using study coupons, with the aim of having 4 or 5 waves of recruitment to reach equilibrium [21]. In total, we approached 1662 participants. However, 36 of the 1662 (2.2%) were not eligible after the initial screening process, resulting in the final sample size of 1626.

### Data collection: Risk behaviors and blood specimens

The data collection was conducted from August 2012 to April 2013. Recruitment sites in each province were selected based on the information from provincial HIV/drug programs that worked with the drug users and were aware of places commonly accessible to PWUDs. The main criterion for recruitment sites was that they provide a space to ensure the privacy and confidentiality of the participants. After oral informed consent was received from the participants, the interviews were conducted by gender-matched interviewers (e.g., male interviewers interviewed male participants) and were followed by a request for a blood sample. The interviewers used a questionnaire to collect data on socio-demographic characteristics, drug-taking behaviors, types and frequency of drug use and HIV-related risk behaviors. The questionnaire was based on a small-scale survey of drug users in Cambodia in 2006 and on pre-testing [22]. The interviews lasted approximately 30 min.

A 5-ml blood sample was drawn and kept in a tube with an anti-coagulant to prevent the blood from clotting. At the end of each day, the blood samples were sent to a laboratory at a Voluntary Counseling and Testing Center (VCT) near the study recruitment site.

### Monitoring and HIV testing and quality control

The interviewers and supervisors were selected from among those who had experience working with these key populations. Both the interviewers and supervisors were trained for 3 days in Phnom Penh on the recruitment process, informed consent procedures and the questionnaire-based interview procedure. The supervisors were responsible for ensuring that RDS sampling was properly performed, the questionnaires were properly completed, and the informed consent process was strictly followed to ensure that the participants could refuse or withdraw from the study at any time.

At VCT, HIV testing was performed using 2 rapid tests. First, blood samples were tested with Determine HIV 1/2 (*Alere HIV*). Specimens that were reactive in the first test were retested with HIV 1/2 Stat-Pak (*Chembio Diagnostic System, Inc*). This standard serial testing algorithm has been used by the national HSS for groups that have an HIV prevalence greater than 10% [2]. Then, the sera were prepared and stored before being sent to the NCHADS laboratory for quality control and storage.

All HIV-positive specimens plus a randomly selected 10% of negative specimens were tested for quality control. Serial testing was performed by the NCHADS laboratory using two ELISA tests (*Vironostika, BioMérieu; and Murex 1.2.0, Murex DiaSorin Biotech*). The Vironostika test was used first. If the result was non-reactive, the test was considered HIV negative. If the Vironostika test was reactive, the results were confirmed via the Murex test.

### Statistical analysis

HIV prevalence estimates were calculated using the RDS analysis tool RDSAT 7.1 [23]. RDSAT was developed to minimize biases associated with the social network referral process by weighting the respondents’ probability of being recruited into the RDS sample and recruitment patterns [21]. Individual weights generated with RDSAT for HIV status were imported into STATA to adjust for the RDS sampling process [24]. Weighted bivariable analysis and multivariate logistic regression (SVY) were used to identify factors associated with HIV prevalence. Potential confounding factors, regardless their significant level, and factors that were associated with HIV infection in the bivariable analysis at *p* <  0.20 [25] were included in the multivariate logistic regression; the included factors were sex, age group, provincial region, marital status, education level, drug use type, number of paid sex partners, and duration of drug use.

## Results

### Demographic characteristics of PWUDs

Of the 1626 respondents, approximately 7.3% were PWIDs. Most of the PWUDs were men (82.0%), while women represented approximately 18.0%. The median age of the PWIDs was 28.0 years, with an interquartile range (IQR) of 24–32 years; the median age for the non-PWIDs was 24.0 years (IQR: 20–29 years). For both groups, the women were approximately 2–3 years older, and the PWIDs tended to be slightly older than the non-PWIDs **(**Table 1**)**. Approximately half of the participants were un-married (i.e., single, widowed, or divorced). The median number of years of schooling was 6.0 (IQR: 3–8 years) for the PWIDs and 7.0 (IQR: 5–9 years) for the non-PWIDs. More than 40% of the PWIDs reported either living with their parents or spouse, while close to 75% of the non-PWIDs reported similar living arrangements. The PWID group was more likely to live with friends (15.3% vs. 7.8%).

**Table 1:** Socio-demographic characteristics of PWUD

Variables	PWID = 119						Non PWID = 1507					
	Men		Women		Total		Men		Women		Total	
	Freq.	%	Freq.	%	Freq.	%	Freq.	%	Freq.	%	Freq.	%
Regional provinces^a^												
Low risk provinces	23	22.3	2	12.5	25	21.0	745	61.9	73	26.8	818	55.4
High risk provinces	80	77.7	14	87.5	94	**79.0**	459	38.1	199	73.2	658	**44.6**
Age, median (IQR)^b^, years	28 (24–31)		30 (26–34)		28 (24–32)		23 (20–28)		27 (22–31)		24 (20–29)	
Age groups												
≤ 20 years old	12	12.1	1	6.3	13	**11.3**	351	29.6	46	17.2	397	**27.3**
21–29 years old	50	50.5	6	37.5	56	**48.7**	594	50.1	138	51.7	732	**50.4**
≥ 30 years old	37	37.4	9	59.2	46	40.0	240	20.3	83	31.1	323	22.3
Marital status												
Married	48	47.1	14	87.5	62	52.5	472	39.5	225	83.0	697	47.5
Non-married	54	52.9	2	12.5	56	47.5	723	60.5	46	17.0	769	52.5
Education, median (IQR), years	7 (3–9)		3 (1–6)		6 (3–8)		8 (6–10)		4 (0–7)		7 (5–9)	
Education level												
0–6 years in school	49	50.0	3	18.7	62	54.4	745	65.3	71	26.8	591	42.0
≥ 7 years in school	49	50.0	13	81.3	52	45.6	397	34.7	194	73.2	816	58.0
Report current living places												
Parent	36	35.3	1	6.2	36	**30.5**	708	59.3	42	15.5	763	**51.0**
Spouse	15	14.7	4	25.0	19	16.0	272	22.8	56	20.7	332	22.2
Friend	15	14.7	4	25.0	18	15.3	60	5.0	54	19.9	117	7.8
Street	5	4.9	0	0.0	5	4.3	15	1.3	20	7.4	38	2.5
Others	31	30.4	7	43.8	40	33.9	139	11.4	99	36.5	245	16.5

^a^High risk provinces: Sihanoukville, Phnom Penh, Battambang, Banteay Meanchey

Low risk provinces: Kampong Speu, Kampong Cham, Prey Veng, Svay Rieng, Siem Reap

^b^*IQR* interquartile range

### Drug use behavior among PWUDs

The median duration of drug use for the PWIDs was 10.0 years (IQR: 5–13 years), compared with 4.0 years (IQR: 1–7 years) for the non-PWIDs **(**Table 2**)**. Less than 20% of the PWIDs first started using drugs by injection; the rest mainly began by smoking and sniffing drugs. Both groups (91%) reported first starting drug use with friends and peers. Frequently, they were first introduced to drugs by their friends (85.9%), followed by self-initiation (8.5 and 6.6% for PWIDs and non-PWIDs, respectively). The drugs that the PWIDs most commonly reported using in the past 12 months were ice/amphetamine (78.0%) and heroin (61.9%), while the non-PWIDs reported using ice/amphetamine (81.5%) and yama (47.1%), a pill containing methamphetamine. Moreover, close to 60 and 38% of the PWIDs and non-PWIDs, respectively, reported having used at least 2 drugs in the past 12 months. In addition, 35.6% the PWIDs reported having shared needles and syringes in the past month.

**Table 2:** Drug taking risk exposures among PWUDs

Variables	PWID = 119						Non- PWID = 1506					
	Men		Women		Total		Men		Women		Total	
	Freq.	%	Freq.	%	Freq.	%	Freq.	%	Freq.	%	Freq.	%
Duration of drug use, median (IQR)	10 (5–13)		10 (4–12)		10 (5–13)		4 (1–8)		3 (1–6)		4 (1–7)	
Duration of any drug use					*n* = 117						*n* = 1468	
Users ≤12 months	7	6.9	3	20.0	10	8.6	303	25.2	82	30.7	385	26.2
Users > 12 months	95	93.1	12	80.0	107	**91.4**	898	74.8	185	69.3	1083	**73.8**
Methods PWUDs used drugs at the first time												
Smoking	74	72.6	14	82.5	87	**73.7**	1158	96.7	254	93.7	1440	**96.1**
Sniffing/drinking	8	7.8	0	0	8	6.9	35	2.9	15	5.5	52	3.5
Injecting	20	**19.6**	2	12.5	22	**18.6**	4	0.3	2	0.7	6	0.4
Persons PWUDs used drug with at the first time												
Friends	89	87.4	13	81.3	103	**87.3**	1141	95.2	202	74.5	1370	**91.3**
Sweethearts/spouses/relatives	8	7.8	2	12.5	10	8.4	26	2.2	47	17.3	73	4.9
Alone	4	3.9	0	0.0	4	3.4	29	2.1	14	5.2	43	2.9
Others	1	0.9	1	6.2	1	0.9	3	0.3	8	3.0	15	0.9
Persons who first introduced you to use drugs												
Friends	85	83.3	12	75.0	98	**83.0**	1079	90.0	189	69.7	1292	**86.1**
Myself	8	7.8	2	12.5	10	**8.5**	73	6.1	22	8.1	99	**6.5**
Sweethearts/spouses/relatives	8	7.8	2	12.5	10	8.5	44	3.7	49	18.1	76	5.1
Others	1	1.0	0	0.0	0	0.0	3	0.2	11	4.1	34	2.3
Types of illicit drug used in the past 12 months												
Ice/amphetamine	79	77.5	14	87.5	92	**78.0**	998	82.9	203	74.6	1228	**81.5**
Yama (pill of methamphetamine)	30	29.4	7	43.8	37	**31.4**	557	46.3	148	54.4	710	**47.1**
Marijuana	16	15.7	1	6.3	17	14.4	140	11.6	13	4.8	153	10.2
Heroin	59	57.8	12	75.0	73	**61.9**	33	2.7	9	3.3	42	2.8
Ecstasy	4	3.9	3	18.8	7	5.9	54	4.5	26	9.6	80	5.3
Others (including inhalant)	13	8.1	1	1.5	19	16.1	149	12.4	17	6.3	136	5.3
Reported uses at least 2 drugs in past 12 months	58	56.8	12	75.0	70	59.3	455	37.9	107	39.4	562	38.1
Last injecting drugs with re-used syringes and needles (*n* = 119)	**–**	**–**	–	–	38	31.9	–	–	–	–	–	–
Reported sharing needles and syringes when injecting drugs in past month (*n* = 59)	**–**	**–**	–	–	21	35.6	–	–	–	–	–	–

### Sexual behavior among PWUDs

More PWIDs (94.1%) had had sex in their lifetimes than non-PWIDs (89.7%). The women in both groups reported more sexual activity than the men **(**Table 3). Among those who had ever had sex in their lifetime, approximately three-quarters of both groups reported “being paid and paying for sex” in the past month. The women reported a higher proportion of paid sex than the men among both the PWIDs (85.8%) and the non-PWIDs (89.4%). However, reports of always using condoms during paid sex did not exceed 50% among the PWIDs but were greater than 60% among non-PWIDs. Condom use among the women in the PWID group was as low as 25%. In addition, reports of condom use with regular partners (spouses, intimate partners, cohabiting partners) was as low as ≤30% in both groups.

**Table 3:** Sexual risk exposures to HIV among PWUDs

Variables	PWID = 113						Non- PWID = 1335					
	Men		Women		Total		Men		Women		Total	
	Freq.	%	Freq.	%	Freq.	%	Freq.	%	Freq.	%	Freq.	%
Report of ever had sex	96	93.2	16	100.0	112	94.1	1063	88.6	256	94.5	1319	89.7
Age at first sex, median (IQR), years	17.5 (17–18)		18 (16–20)		18 (17–20)		18 (17–20)		18 (16–19)		18 (16–19)	
Age at first sex in years												
≤ 18 years old	73	76.0	10	62.5	83	**74.1**	557	52.5	166	64.6	723	**54.9**
> 18 years old	23	24.0	6	37.5	29	**25.9**	504	47.5	91	35.4	595	**45.1**
Ever had sex in the past month among those who ever reported sex	53	56.4	7	43.4	60	54.6	713	67.5	206	80.2	919	70.0
Report number of paid and paying sex partner in the past month					***n*** **= 99**						***n*** **= 1181**	
No sex partner	73	**79.3**	1	**14.2**	74	**74.8**	854	**82.1**	15	**10.6**	869	**73.6**
≤ 2 sex partners	11	12.0	3	42.8	14	14.1	141	13.6	45	31.9	186	15.8
≥ 3 sex partners	8	8.7	3	42.8	11	11.1	35	4.3	81	57.5	126	10.6
Always condom use with paid and paying sex partners in the past 12 months	44	43.1	4	25.0	48	**40.6**	746	62.1	165	60.7	911	**61.8**
Condom use in last sex in exchange for money	52	86.7	6	85.7	58	86.6	683	88.4	136	88.9	819	88.4
Always condom use with regular partners in the past 12 months	17	30.4	4	30.8	21	30.4	191	25.3	36	17.2	227	23.6
Condom use last time when had sex with regular partner	29	46	8	61.4	37	49	409	50.7	96	43.1	505	49.1

### HIV prevalence among PWUDs

As shown in Table 4, the overall HIV prevalence among PWUDs was 5.1% [95% CI: 4.1–6.2]. The HIV prevalence among PWIDs was 24.8% [95% CI: 7.3–39.9]; among non-PWIDs, it was 4.0% [95% CI: 2.7–5.5]. The HIV prevalence among female PWIDs (37.5, 95% CI: 10.9–64.1) was higher than that among male PWIDs (22.5%, 95 CI: 14.0–30.9). A similar pattern was found among female non-PWIDs (11.5, 95% CI: 7.9–15.7) and male non-PWIDs (1.5, 95% CI: 0.8–2.2).

**Table 4:** HIV prevalence among PWUDs

PWID			Non-PWID		
Men	Women	Total	Men	Women	Total
% (95% CI)	% (95% CI)	% (95% CI)	% (95% CI)	% (95% CI)	% (95% CI)
22.5 (14.0–30.9)	37.5 (10.9–64.1)	24.8 (7.3–39.9)	1.5 (0.8–2.2)	11.5 (7.9–15.7)	4.0 (2.7–5.5)

### Factors associated with HIV infection in the logistic regression

The details of the bivariate analysis are presented in Table 5. The following factors were significantly associated with HIV: higher-risk province group [odds ratio (OR) = 5.2, 95% CI: 2.6–10.5] (with the lower-risk province group used as the referent group); female (males as the reference) (OR = 5.6, 95% CI: 3.0–10.6); and older age groups (aged ≤20 years as the reference) 21–29 (OR = 21.2), 30 and older (OR = 78.5). Low education level (secondary/higher education as the reference) and married PWUDs (non-married as the reference) were associated with HIV infection. The PWIDs had approximately 5 times higher odds of HIV infection than the non-PWIDs (OR = 4.6, 95% CI: 2.3–9.2). Furthermore, having had ≥2 paid sex partners in the past month and having used drugs for ≥12 months were significantly associated with HIV infection, with OR = 3.5 (95% CI: 1.6–7.5) and OR = 2.7 (95% CI: 1.3–5.6), respectively. The associations for other covariates, including reported consistent condom use with paid sex partners in the past 12 months and multiple drug use, were not statistically significant.

**Table 5:** Risk factors associated with HIV in bivariate and multivariable logistic regression among drug users

Variables	*N* = 1583		Total sample (*N* = 1186)	
	OR (95% CI)	*P* value^b^	AOR^a^ (95% CI)	*P* value^b^
Provincial group^a^				
Low risk provinces	Referent		Referent	
High risk provinces	5.2 (2.59–10.56)	< 0.001	1.9 (0.78–4.79)	0.154
Sex of drug users				
Men	Referent		Referent	
Women	5.6 (2.95–10.59)	< 0.001	7.8 (3.00–20.35)	**< 0.001**
Age group in years				
≤ 20	Referent		Referent	
21–29	21.2 (2.73–164.16)	0.003	10.3 (1.20–89.39)	**0.033**
≥ 30	78.5 (10.53–584. 18)	< 0.001	36.4 (3.59–369.36)	**0.002**
Marital status				
Non-married	Referent		Referent	
Married	2.9 (1.37–6.01)	0.005	0.48 (0.17–1.47)	0.205
Education level				
≤ 6 years (Primary)	2.4 (1.22–4.68)	0.011	0.92 (0.37–2.27)	0.866
> 6 years (Secondary and higher)	Referent		Referent	
Drug use type				
Non-PWID	Referent		Referent	
PWID	4.6 (2.31–9.19)	< 0.001	4.1 (1.53–10.96)	**0.005**
Number of paid and paying sex partners In past month				
< 2 partners	Referent		Referent	
≥ 2 partners	3.5 (1.64–7.48)	0.001	1.1 (0.42–2.47)	0.961
Duration of using drugs				
≤ 12 months	Referent		Referent	
> 12 months	2.7 (1.27–5.63)	0.010	4.0 (1.38–11.35)	**0.010**
Consistent condom use with casual				
partner in the past 12 months				
Yes	Referent			
No	1.1 (0.59–2.10)	0.735	–	–
More than one drug use in past 12 months				
One drug	Referent			
More than one drug	1.1 (0.59–2.10)	0.735	–	–

^a^High risk provinces: Sihanoukville, Phnom Penh, Battambang, Banteay Meanchey

^b^The results in this table were weighted

Low risk provinces: Kampong Speu, Kampong Cham, Prey Veng, Svay Rieng, Siem Reap

In the final multivariable model, 4 factors were found to be independently associated with HIV infection: sex (women), older age groups, injected drug use and duration of drug use ≥12 months **(**Table 5). Women had higher odds of HIV infection than men, with an adjusted OR (AOR = 7.8, 95% CI: 3.0–20.4), and participants older than 20 years had a higher odds of HIV infection: age groups 21–29 (AOR = 10.3, 95% CI: 1.2–20.4) and age group ≥30 (AOR = 36.4, 95% CI: 3.6–369.4). Injected drug use remained a strong predictor of HIV infection, with AOR = 4.1 (95% CI: 1.5–10.9). Additionally, the longer a PWUD had used drugs, the higher their odds of HIV infection, with AOR = 4.0 (95% CI: 1.4–11.4).

## Discussion

This study reported a high prevalence of HIV among PWIDs and a large proportion of non-PWIDs, especially ATS users. Most of the PWUDs were sexually active, and they indicated a high proportion of paid sex and low consistent condom use, especially among PWIDs. The main predictors of HIV infection included female sex, injected drug use, older age and drug use for ≥12 months.

In our study, the HIV prevalence among PWIDs was approximately 25%, which is similar to the prevalence levels for Cambodia reported a few years ago by Mathers et al., who reviewed and estimated the global HIV prevalence among drug users and the size of the HIV-infected drug user population and injecting drug user population by country [26].

Despite the lower prevalence of HIV (4%) among non-PWIDs, the large non-PWUDs population (90%) remains a public health and a matter of social concern for several reasons. First, recent reports may indicate shifting patterns of HIV infection in Cambodia from non-PWIDs to PWIDs. Our findings indicated that less than 20% of PWIDs initially injected drugs. Additionally, a report from the KHANA Drop-In Center (DiC) showed that less than 5% of PWUDs who visited the center were originally injected drug users. However, in one year of the DiC implementation, approximately 3% of its visitors converted from non-PWIDs to PWIDs *(Personal communication with DiC)*.Therefore, these raise a concern what prompts PWUDs to become PWIDs and the possibility that that HIV transmission among non-PWIDs in Cambodia could increase in the future due to their related risk behaviors and the overlapping social and sexual networks among PWUDs [16, 27]. Additionally, the high proportion of transactional sex among female PWUDs in this study, particularly among non-PWID women, indicates the possibility that infection could be transmitted to non-injected drug users and then to the general population through transactional sex. Consequently, these factors potentially contribute to an increase in overall HIV prevalence in Cambodia. Therefore, it is important for HIV and drug use intervention programs to significantly target these high-risk groups.

Needle and syringe sharing among PWIDs remains high, and implements are often shared with little or no cleaning. Given that sterile needle and syringes programs (NSPs) are a key component of harm reduction and HIV prevention efforts [15, 28–30], more targeted interventions for PWIDs should be implemented continuously, without law enforcement barriers, to ensure access to adequate supplies of clean needles and syringes. Prior studies estimated that interventions (i.e., opioid substitution, needle exchange and antiretroviral therapy) that attain at least 60% coverage could cut future infections among PWIDs roughly in half, thereby decreasing the HIV epidemic among PWIDs [28]. However, the program report suggested that NSPs had very low coverage - only 16% of PWIDs reported accessing NGO drop in centers in the past 12 months [31]. Further research should include NSP assessments to improve program interventions. Additionally, the large proportion of non-PWIDs, especially ATS users, is worrisome and very challenging for prevention efforts since effective prevention strategies for ATS use has little evidence base. A study of an integrated HIV and drug prevention program with conditional cash transfer was conducted in Cambodia to test the use of behavioral interventions among ATS users for improved prevention measures for this group; the results have not been published [32]. However, another formative research among study by *Carrico* et al. to reduce the risk of ATS among entertainment workers using the conditional cash transfer with behavioral intervention found the mixing results [33].

The findings of a higher risk of HIV among women who use drugs are consistent with the literature, which highlights issues associated with increased vulnerability for women (e.g., child care, concomitant sex work, lack of access to health care, mental and physical health problems, reproductive health issues, sexually transmitted infections, stigma, and violence) [8, 30]. Beyond these issues, female PWUDs with overlapping risk behaviors, such as transactional sex and drug use, often have less power to negotiate safe sex practices [8, 17, 34]. According to Azim et al., female PWUDs who were sex workers were more likely than non-drug using sex workers to engage in street-based sex work, which is associated with high-risk sex and heightened levels of violence due to different types of partners [30].

Given the high HIV prevalence among drug users with a high frequency of paid sex partners, focused intervention to assist this sub-group is an important public health goal in Cambodia. Evidence in prior studies suggests that female PWIDs who are sex workers constitute a “bridge population” [35] that can lead to a the spread of HIV epidemics from PWIDs to heterosexual populations [30, 36].

Multiple structural and behavioral interventions specifically designed for female PWUDs have been implemented globally with tailor-made interventions adapted to women’s specific needs [30]. For example, a study investigating the effectiveness of HIV/STI safer sex skill-building groups for women found that these groups improved safer sex practices compared with standard HIV/STI education [37]. However, many women still struggle alone to change risky behaviors with their partners, and in these situations, it may be more effective to engage couples in harm reduction interventions [30]. Also, access to legal and heath care supports should be addressed in the multiple structural interventions.

Comprehensive interventions that address individual and socio-environmental factors are more effective than a single intervention alone [29]. Such interventions need to include many of the interventions outlined above and should also outline steps for improving understanding and sensitivity among professionals who interact with PWUDs (e.g., health care workers or NGO staff) [30]. This is particularly the case with law enforcement. As a recent study surmised, “Fear of accessing harm reduction and health services and police’s negative attitudes and practices towards key populations present major barriers to HIV prevention efforts in Cambodia” [19, 38]. Efforts to reduce the fear of retaliation and/or stigma may help improve the effectiveness of broader intervention programs.

This study has several limitations**.** First, self-reports of sensitive information (i.e., drug use and risky sexual behaviors) and social desirability may result in the under-reporting of actual information. Second, the lifetime and 12-month recalls used in some questions may have caused recall issues. Third, although RDS was used to recruit a representative sample, we are not sure how well the seeds were represented and referred or how many PWUDs did not participate in the study (mostly PWIDs, due to self-stigma or discrimination), and we do not know the different characteristics and HIV-related risk behaviors of those who did not participate in the study. Given these factors, we may have underestimated the HIV prevalence among PWIDs - especially female PWIDs, given the small samples - and weakened the association between the predictors and the HIV prevalence. Despite these limitations, this is the first ever large-scale survey conducted in Cambodia among PWUDs using the RDS method and involving many key stake- holders’ involvement (NCHADS, NACD, UNAIDS, NGOs). It provides useful and informative findings to guide HIV and drug use program planning and policy for future interventions.

## Conclusion

HIV prevalence among PWUDs remains high especially among PWIDs and women. Harm reduction programs, such as NSP, must be improved in scope and scale. Innovative strategies are needed to reach sub-groups of PWUDs, especially women who inject drugs. Furthermore, the large proportion of non-PWIDs, especially ATS users, should not be ignored; combined HIV prevention and harm reduction programs should integrate ATS users.

## Abbreviations

ART: Anti-retroviral therapy;
ATS: Amphetamine-type stimulants (ATS)
DiC: Drop-In Center
HSS: HIV Sentinel Surveillance
NACD: National Authority for Combating Drugs
NCHADS: National Center for HIV/AIDS, Dermatology and STIs
NSP: Needles and Syringes Program
PWIDs: People who inject drugs
PWUDs: People who use drugs
RDS: Respondent-driven sampling
VCT: HIV Voluntary Counseling and Testing Center
